# Bedsharing may partially explain the reduced risk of sleep-related death in breastfed infants

**DOI:** 10.3389/fped.2022.1081028

**Published:** 2022-12-13

**Authors:** Melissa Bartick, Michal Young, Adetola Louis-Jacques, James J. McKenna, Helen L. Ball

**Affiliations:** ^1^ Department of Medicine, Mount Auburn Hospital/Beth Israel Lahey Health, Cambridge, MA, United States; ^2^ Department of Medicine, Harvard Medical School, Boston, MA, United States; ^3^ Department of Pediatrics and Child Health, Howard University College of Medicine, Washington, DC, United States; ^4^ Department of Obstetrics and Gynecology, University of Florida Health, Gainesville, FL, United States; ^5^ Department of Anthropology, Santa Clara University, Santa Clara, CA, United States; ^6^ Department of Anthropology, University of Notre Dame, South Bend, IN, United States; ^7^ Department of Anthropology, Durham Infancy & Sleep Centre, Durham University, Durham, United Kingdom

**Keywords:** sudden infant death, breast feeding, infant care, infant, infant mortality

## Introduction

Studies have shown that breastfeeding is associated with a lower risk of Sudden Infant Death Syndrome (SIDS) (1, 2) and Sudden Unexpected Infant Death (SUID) (3), and the association with SIDS is thought to likely be causal (2). In their 2011 meta-analysis on breastfeeding and SIDS risk, Hauck et al. proposed possible mechanisms, including increased arousability of the infant and immunoglobulins and cytokines in breast milk that may protect infants during the vulnerable period of SIDS, particularly considering that SIDS is often preceded by a minor infection (2). Breastfed infants are more easily aroused from active sleep than formula fed infants at 2–3 months of age (4). Increased brain myelination seen in breastfed infants (5) may affect SIDS risk (6). These mechanisms are plausible for some deaths, but more evidence is needed.

The evidence for a protective association between breastfeeding and SIDS was consistent enough across many observational studies for the American Academy of Pediatrics (AAP) to recommend “human milk feeding” in its 2022 guidelines to prevent SIDS (7). However, it is unclear what aspects of breastfeeding are conferring this protection, and if it is breastfeeding or human milk that is responsible.

## Bedsharing

Bedsharing (defined here as sharing an adult bed) facilitates breastfeeding and is associated with more night-time breastfeeding (8). However, the AAP and other organizations have long recommended that parents avoid any bedsharing in order to prevent SIDS (9), due to concerns that bedsharing may cause SIDS and other sleep-related infant death. Sleep-related death, or SUID, includes SIDS plus accidental suffocation (ICD-10 code W75), and “ill-defined” death (ICD-10 code R99). In its 2022 recommendations, the AAP states that “on the basis of evidence, the AAP is unable to recommend bed sharing under any circumstances” (7). There is widespread consensus that co-sleeping (defined here as a sharing any sleep surface) with hazardous circumstances increases the risk of death. However, not all experts agree that bedsharing is universally unsafe (10). Authorities in Spain, the United Kingdom, and Norway are no longer advising against bedsharing when no hazards exist (11–13).

Co-sleeping is associated with an increased risk of sleep-related death in certain hazardous circumstances. Hazardous circumstances include sofa-sharing, co-sleeping in a chair, infant tobacco exposure, co-sleeping with an adult impaired by alcohol, and co-sleeping with a low-birthweight or preterm infant (7, 10, 14).

The AAP cited a 2013 study by Carpenter et al. as evidence for its recommendation against bedsharing (15). This study showed an increased risk of death in the absence of hazardous circumstances, but has been criticized for using an unrealistic comparator group for co-sleeping, among other reasons (10). A 2014 study by Blair et al. of 400 SIDS cases from the 1990s and mid 2000s found no increased risk of bedsharing in the absence of hazards (14). Other analyses of the literature (10–12) have not drawn the same conclusions as the AAP and its statistician who reviewed these two studies (16). The Blair et al. study found bedsharing in the absence of hazards was protective against SIDS in infants older than 3 months (14). In addition, many populations with high bedsharing rates have low rates of sleep-related death (17, 18), and high population levels of hazardous risk factors may account for high levels of death in those populations where bedsharing rates are high (17). The most recent case control study from New Zealand of 132 SUID cases showed that co-sleeping was only a significant risk when parents smoked (19). A subsequent publication from the same data set identified alcohol, drugs, and sofa-sharing as other hazards in association with co-sleeping, but they were only significant risks when combined with smoking (20).

Breastfeeding is one of the main reasons given for bedsharing, and getting more sleep is another leading reason (21). Bedsharing has been associated with longer durations with any (22–24) and exclusive (22, 25) breastfeeding. Over 60% of all US mothers bedshare (26), and the proportion of breastfeeding mothers who bedshare is likely to be much higher.

Several physiologic features of bedsharing may be protective against sleep-related death among breastfeeding infants (27). Videographic evidence shows that breastfeeding bedsharing infants rarely sleep prone (27, 28). After feeding, breastfeeding infants roll onto their backs (28). Breastfeeding mothers naturally position their infants with their heads alongside their breasts, encircling the infants with their arms and legs. The mother's arm forms a barrier between the infant's head and the pillow (Prone sleep and pillows are risk factors for sleep-related death.) Both mothers and infants are more arousable when bedsharing (27, 29, 30). They breastfeed more frequently than dyads sleeping separately (8). The bedsharing mother-infant dyad also experience increased sleep synchrony (27). Mothers also perceive an increased ability to be vigilant to infant dangers by bedsharing (31). In addition, routine (planned) bedsharing is not associated with an increased risk of SIDS (32). Accidental suffocation death is extremely rare among breastfeeding bedsharing infants in the absence of hazardous circumstances (10, 33). Growing anthropologic evidence suggests that breastfeeding with bedsharing is the human evolutionary norm (34).

A videographic study of bedsharing families showed that 71% of formula-feeding infants had their heads at the level of the mother's face, with their heads on pillows or between the parents’ pillows, but every breastfed infant's head was at the level of the mother's chest (28).

Interestingly, the study by Blair et al. only showed a protective effect of breastfeeding among infants sleeping alone. Perhaps this is not surprising as the strong association between breastfeeding and bedsharing (23, 35) would minimize the differences in any group comparison of bedsharers between cases and controls. In addition, due to the large drop-off in breastfeeding rates by 6 weeks in the UK (36), many of the formula fed infants in the control group may have been breastfed, and may have bedshared in a way similar to breastfed infants, further minimizing the difference between the two groups. The 2017 Thompson et al. meta-analysis of case-control studies on breastfeeding and SIDS risk included 2,259 SIDS cases and found that breastfeeding was associated with a markedly decreased risk of SIDS in a dose-dependent fashion (1). In that analysis, these authors controlled for co-sleeping but not all the hazards in this environment (e.g., alcohol or drug exposure), and they were unable to control for socioeconomic status. Neither of these studies explored breastfeeding prevalence in the hazardous versus non-hazardous bedsharing environments. It might be that breastfeeding affords protection to infants when bedsharing in the absence of any hazards. Larger case-control studies are needed to investigate this but may no longer be viable given the welcome reduction in SUID deaths in the US and UK since the 1990s. Detailed national mortality data on SUID combined across countries would shed more light on these associations, as can physiologic data.

In addition, videographic data on infant positioning in bedsharing among breastfeeding and formula feeding mothers and data for infant arousability with formula suggest that it is plausible that formula feeding confers excess risk in the absence of hazards compared with breastfeeding. This risk might be mitigated with education for all parents on safe bedsharing positioning.

## Bedsharing as a confounder

A confounder is a variable that distorts the association between the exposure of interest and the outcome, because it is associated with both the exposure and the outcome. Bedsharing, when defined as sharing a bed, confounds the association between breastfeeding and sleep-related death because it is associated with both breastfeeding (the exposure) and death (the outcome). While it has frequently been stated that co-sleeping carries a higher risk of SIDS, bedsharing clearly has some protective effects in the absence of hazardous circumstances. In the association between breastfeeding and sleep-related death, the role of bedsharing in the presence of hazards is complex. Preterm/low birth weight infants, infants with tobacco exposure, and infants sleeping next to an adult with alcohol exposure, are less likely to be breastfeeding (37–40). We can hypothesize that bedsharing might be more likely to occur in positions that are unsafe in these infants (e.g., on pillows). Sofa-sharing or chair-sharing are likely unrelated to breastfeeding, so they would not be confounders. Bedsharing could also have a multiplicative interaction with many of the hazards (e.g., smoking, alcohol) to magnify risk. For smoking, one mechanism of magnified risk is that bedsharing infants of smokers have greater exposure to tobacco than non-bedsharers, as measured by urine cotinine levels (41).

Breastfeeding is a complex behavior which differs substantially from feeding human milk from a bottle. No studies have examined whether bottle-feeding human milk would be associated with a lower risk of SIDS, particularly in the absence of bedsharing, and such a study would be very difficult to conduct given both the rare outcome of sleep-related death, and the fact that this feeding mode is uncommon (5.6% of US infants are fed by exclusively pumped milk) (42).

It is not currently possible to separate the effects of bedsharing and other breastfeeding behaviors from the effects of the milk itself. We thus do not know how much of the protective association of breastfeeding on sleep-related infant death is actually due to bedsharing and other behavioral effects, and not just due to the milk itself.

The role of bedsharing in sleep-related infant death is clearly nuanced. There appears to be a split risk, with increased risk in the presence of certain hazards, but bedsharing most likely has a net protective effect when associated with breastfeeding, in the absence of hazards.

## Discussion

Considering bedsharing as a protective confounder has significant implications. Separating a breastfeeding infant from their mother would deprive the infant of the established protective effects of bedsharing. Currently, it is common to see advice such as “the safest place for a baby to sleep is in a crib near your bed” (43). Assuming no hazardous circumstances, the safest place for a breastfeeding infant may be in bed with their mother. This possibility is sufficient to at least question the long-standing advice of separate sleep until more research is conducted. Public health advice should allow for flexibility. For example, low-risk families should plan for separate sleep when they consume alcohol.

Separating an infant from their mother may impact duration of breastfeeding, which itself has health implications. Not only do multiple studies show a strong association between bedsharing and breastfeeding duration, the additional evidence on bedsharing and increased frequency and length of feeds with bedsharing compared to solitary sleep (8) together suggest a causal relationship between bedsharing and breastfeeding duration.

If promoting solitary sleep results in shorter durations of breastfeeding, this has significant implications for reducing the risk of disease in mothers and children. Promoting separate sleep, even in the absence of hazards, may have a negative and sizeable impact on population health. For example, population rates of maternal cardiovascular disease and stroke (44), breast cancer (45), childhood obesity (46), infant gastrointestinal infection, and hospitalization for lower respiratory tract infection are impacted by premature weaning (47), which may be increased by promotion of solitary sleep. Cognitive function in children is also impacted by breastfeeding duration and could have substantial economic implications (48–50). Finally, because SIDS occurs in a dose-response fashion with respect to breastfeeding duration (1), it is possible that promotion of solitary sleep may indirectly increase SIDS rates.

In sum, bedsharing has known protective effects among breastfeeding infants and confounds the protective association between breastfeeding and sleep-related infant death. It is time to revisit the historic conclusions from the literature that bedsharing is responsible for sleep-related death, and understand bedsharing's more nuanced role in light of what we know about its physiology among breastfeeding infants and the basic principles of epidemiology.
